# An unexpected benefit from *E. coli*: how enterobactin benefits host health

**DOI:** 10.15698/mic2018.10.653

**Published:** 2018-09-27

**Authors:** Aileen K. Sewell, Min Han, Bin Qi

**Affiliations:** 1 Department of Molecular, Cellular and Developmental Biology, University of Colorado, Boulder, CO USA.; 2 Howard Hughes Medical Institute.

**Keywords:** commensal, microbiome, microbiota, holobiont, C. elegans, siderophore, iron deficiency

## Abstract

Iron plays many critical roles in human biology, such as aiding the transport of oxygen and mediating redox reactions. Iron is essential for life, yet little is known about how iron is taken up into mitochondria to impact the labile iron pool. Iron deficiency is one of the most prevalent human nutrient-deficiency diseases in the world and is a major cause of anemia that affects >25% of the world’s population, but unfortunately the current treatment (oral iron supplementation) is inefficient and has many side effects. A greater understanding of iron uptake, and discovery of molecules that aid in this process, may lead to more effective treatments for iron deficiency. In this study, we uncovered a unique and surprising role for an *Escherichia coli*-produced siderophore enterobactin (Ent) that facilitates iron uptake by the host, observed in both *C. elegans* and mammalian cells. Although siderophores are well-known Fe^+3^ scavengers, this activity has previously been described to only benefit iron acquisition by bacteria, not the host. This unexpected function is dependent on the binding of Ent to the host’s ATP synthase α-subunit but is independent of other subunits of the ATP synthase. This finding marks a major shift regarding the role of this siderophore in the “iron tug-of-war” paradigm, which is often used to describe the fight between the bacteria and the host for this essential micronutrient. Instead, this study presents *E. coli* as a commensal “friend” that provides a molecule that supports the host’s iron homeostasis. This work reveals a novel, beneficial role of a bacteria-generated molecule in aiding the host’s iron homeostasis, and points to surprising new benefits from commensal bacteria.

## INTRODUCTION

**Figure 1:**
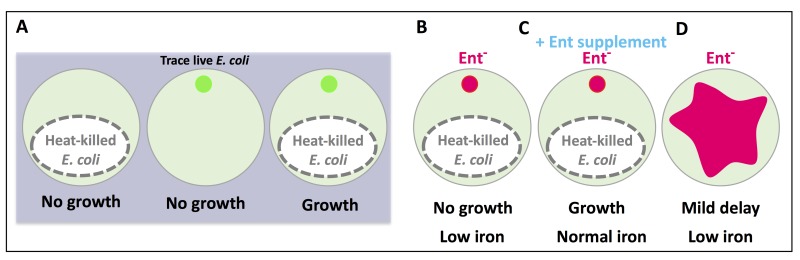
FIGURE 1: A cartoon depiction of the feeding assay used in this study. The phenotypic outcome of each feeding condition is listed below each plate. **(A)** Heat-killed *E. coli* is not sufficient to support worm growth, nor is a trace amount of live *E. coli*, but when they are combined, worm growth is supported. **(B)** *E. coli* mutants deficient in enterobactin (Ent) synthesis did not support worm growth and reduced host iron. **(C)** Supplementation of Ent to Ent-deficient *E. coli* rescued worm growth and iron. **(D)** Feeding abundant Ent-deficient *E. coli* to worms resulted in a mild growth delay and low iron. The small circles represent trace, live *E. coli* (green: wild type, pink: Ent^-^). The pink shape in (D) represents a lawn of abundant Ent^- ^*E. coli*.

Extensive studies in the microbiota field have clearly indicated a symbiotic relationship between a multitude of commensal bacteria and their animal hosts. How the host benefits from the individual metabolites produced by commensal bacteria remains a highly attractive problem to investigate. *E. coli* is a highly prevalent bacterial species in the mammalian gut microbiome. In order to identify individual *E. coli*-produced metabolites, among the many, that provide significant benefits to host physiology, we created a highly sensitive assay system to screen an *E. coli* mutant library using the nematode *C. elegans* as the host (Figure 1A). When fed heat-killed bacteria, *C. elegans* did not grow. When fed a trace amount of live bacteria, *C. elegans* also did not grow since there was too little bacterial food. Yet, when a trace amount of live bacteria was added to the plate with heat-killed bacteria, worm growth was restored. The premise of this assay condition is that the heat-killed bacteria provide the bulk of the nutrients while the trace live bacteria more likely function as the microbiota, able to colonize the intestine and perform “commensal” activities that benefit the host. This assay condition is quite distinct from the typical laboratory feeding condition used for*C. elegans* where live bacteria are abundant. In this manner, we tested for mutant *E. coli* that could not support *C. elegans* growth under these limiting conditions.

From the screen, five of the *E. coli* mutants that failed to support normal *C. elegans* growth were defective in enterobactin (Ent) synthesis (Figure 1B). This finding was very intriguing since no previous studies had described a beneficial impact of bacterial Ent on any animals. Ent is a siderophore, one of many iron-scavenging molecules produced by bacteria. Supplementation with Ent fully rescued the growth of worms fed Ent-deficient bacteria (Figure 1C), but no rescue was seen with supplementation of two other siderophores (pyoverdine and ferrichrome). Although Ent was necessary for growth under our assay condition, it was not absolutely required when worms were well-fed on Ent-deficient bacteria (Figure 1D). Worms fed abundant Ent-deficient bacteria had a mild growth delay and low iron phenotype, compared to those fed Ent-rich bacteria. These results likely indicate a necessary role for Ent under nutrient-limiting conditions and a supportive role under nutrient-rich conditions.

Due to the well-known role of Ent in iron binding, we investigated the iron level in worms under our assay conditions. By several methods, we observed that worms fed Ent-deficient bacteria did indeed have decreased iron levels, and this decrease was eliminated by Ent supplementation (Figure 1B-C). Dramatically, we also found that addition of Ent alone was sufficient to raise iron levels in the host, even under iron-poor conditions where worms were treated with CaEDTA, indicating that Ent may make iron more bioavailable to the host, possibly through its binding to Fe^+3^ or trafficking in the host cell. Ent biosynthesis in bacteria is known to be induced by low iron lor repressed under iron replete conditions. When we supplemented live *E. coli *with FeCl_3_, we did observe animal growth defects and these defects were suppressed by Ent supplementation, supporting a repression of Ent production in *E. coli* by the addition of iron and a critical role of Ent in moving iron into animals. This may also suggest that the iron level in the gut of *C. elegans*, under the typical laboratory culturing condition or in the natural soil environment, usually does not reach the height that fully represses Ent production by gut microbiota.

Through an affinity purification screen, we further found that bacterial Ent binds to the α-subunit of the ATP synthase in the host (ATP-1 in *C. elegans*, ATP5A1 in mammalian cells). The ATP synthase is a multi-subunit enzyme that collectively generates ATP in the mitochondria, and the α-subunit plays a key role in this enzyme activity. This finding was another surprise because the ATP synthase α-subunit was never known for such a role seemingly unrelated to ATP production. We found that the ATP synthase α-subunit had a unique ability to bind to Ent and this binding was required for Ent-dependent promotion of host iron level. Interestingly, we found that the ATP synthase α-subunit carried out this role independent of the whole ATP synthase, and independent of binding to ATP.

Through a series of both *in vitro* and *in vivo* ^55^FeCl_3_ uptake assays, we investigated how Ent and its interaction with ATP-1 impacts the iron level in host mitochondria. We found that Ent supplementation increased the iron level in mitochondria, and that increase was dependent on the ATP synthase α-subunit, but not other subunits of the ATP synthase. We also found that this Ent-Fe^+3^ was bioavailable, as indicated indirectly by the increased activities of iron-dependent enzymes in the mitochondria (aconitase and succinate dehydrogenase).

**Figure 2:**
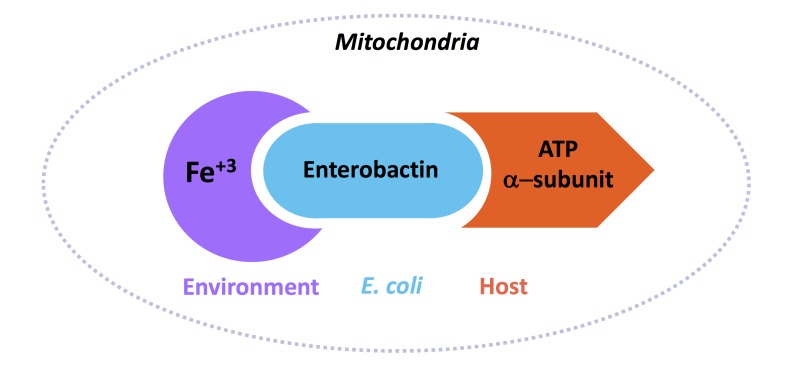
FIGURE 2: A cartoon diagram of the interaction between ATP synthase α-subunit from the host, enterobactin from *E. coli*, and Fe^+3^ from the environment. Together, this complex brings iron to the host mitochondria.

This study led to the model that commensal *E. coli* in the host gut secrete Ent that binds to Fe^+3^ in the environment, Ent-Fe^+3^ then enters the host mitochondria where Ent binds to the ATP synthase α-subunit (Figure 2). The mechanism by which Ent-Fe^+3^ enters the mitochondria is not well-known but may happen by diffusion. Data from our *in* *vitro* mitochondria iron uptake assay may suggest that binding with the ATP synthase α-subunit retains Ent-Fe^+3^ in the mitochondria.

We found that this impact of Ent on host iron was also conserved in assays performed in mammalian cells. It will be very important to investigate the conservation of this role in a multicellular mammalian model, as these findings suggest the tantalizing possibility that enterobactin might serve as a type of therapeutic “helper” to enhance the efficiency of iron supplementation therapy in the treatment of iron deficiency disorder.

In the wild, *C. elegans* come in contact with many different types of bacteria, and there are several different types of siderophores produced by these bacteria. While this study focused on *E. coli *and enterobactin, it will be interesting to investigate if and how other bacteria-generated siderophores might perform a similar function for the host.

While the host intestine provides a rich environment for the proliferation of bacteria, perhaps it is the beneficial roles of those bacteria, such as enterobactin synthesis, that are part of the “give and take” of this symbiotic relationship. Such a hypothesis is already supported by many findings, including our work, that have shown that microbial metabolites have important roles in the host. Further studies like this one will help to unravel the specific roles and underlying mechanisms of this multitude of metabolites.

